# A methodological framework to assess the accuracy of virtual reality hand-tracking systems: A case study with the Meta Quest 2

**DOI:** 10.3758/s13428-022-02051-8

**Published:** 2023-02-13

**Authors:** Diar Abdlkarim, Massimiliano Di Luca, Poppy Aves, Mohamed Maaroufi, Sang-Hoon Yeo, R. Chris Miall, Peter Holland, Joeseph M. Galea

**Affiliations:** 1 University of Birmingham, Edgbaston B15 2TT Birmingham, UK; 2 Department of Psychology, Goldsmiths, University of London, SE14 6NW London, England

**Keywords:** Hand-tracking, Virtual reality, Metaverse, Tracking precision, VR delay

## Abstract

Optical markerless hand-tracking systems incorporated into virtual reality (VR) headsets are transforming the ability to assess fine motor skills in VR. This promises to have far-reaching implications for the increased applicability of VR across scientific, industrial, and clinical settings. However, so far, there are little data regarding the accuracy, delay, and overall performance of these types of hand-tracking systems. Here we present a novel methodological framework based on a fixed grid of targets, which can be easily applied to measure these systems’ absolute positional error and delay. We also demonstrate a method to assess finger joint-angle accuracy. We used this framework to evaluate the Meta Quest 2 hand-tracking system. Our results showed an average fingertip positional error of 1.1*cm*, an average finger joint angle error of 9.6^∘^ and an average temporal delay of 45.0 *ms*. This methodological framework provides a powerful tool to ensure the reliability and validity of data originating from VR-based, markerless hand-tracking systems.

## Introduction

With recent advances in machine learning, mobile computing, and augmented, mixed, and virtual reality (commonly known as extended reality or XR), optical markerless motion-tracking technology has become one of the most cost-effective and easy-to-implement alternatives for recording hand and finger movements across a wide range of applications, (Scheggi, Meli, Pacchierotti, & Prattichizzo, 2015; Voigt-Antons, Kojic, Ali, & Möller, 2020; Khademi et al., 2014). This is in contrast to marker-based hand-tracking, here referred to as ground-truth tracking, which requires individual markers to be placed on each tracked finger/hand and a complicated network of specialized and carefully calibrated optical or magnetic sensors, (Han et al., 2020; Kim et al., 2020; Boian et al., 2002; Schröder, Maycock, Ritter, & Botsch, 2014).

**Fig. 1:**
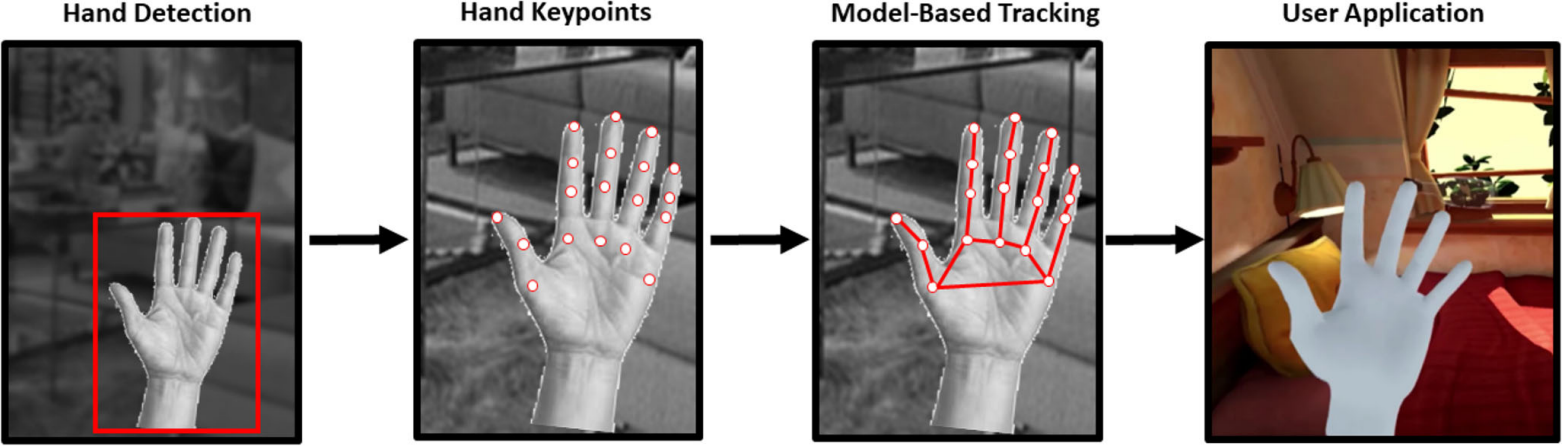
Meta Quest 2 has a multi-stage process for hand-tracking, which includes separate stages for hand detection, key-point identification, and tracking

One popular example of this XR markerless motion-tracking software is the Meta Quest 2 (referred to as Quest, from here on). The Quest 2 hand-tracking system is implemented entirely on the device, without needing a PC, using a multi-stage process to estimate hand pose and finger angles in real time, Fig. 1, see (Han et al., 2020) for more details. The first stage involves correctly identifying whether a hand is present and its location, thus separating the hand from the surrounding objects and background (hand detection). Next, several key points are identified and labeled on the hand and fingers (hand keypoints), which serve as input to an inverse kinematics model of the hand in the next stage (model-based tracking). The estimate of hand pose and finger joint angles is then computed. In the final stage, the output of this model is fed into the user application as position and joint angle data (user application), for example, to display a corresponding virtual hand in a virtual environment.

The use of markerless motion-tracking is spreading rapidly amongst researchers from a wide range of scientific fields due to the relatively small number of hardware components involved, the well-established technology, and reduced costs. For example, in robotics, accurate human hand and finger tracking data are used to safely teach robots to grasp various objects and to perform complex interactions in a simulation environment, before being deployed in the real world (Abdlkarim et al., 2021). In the field of human–computer interaction, markerless hand-tracking allows for research into more natural ways of interacting with virtual objects in immersive environments (Ortenzi et al., 2022). In psychology and neuroscience, optical markerless tracking has the potential to enable research into a wide variety of areas, such as tool use, social interactions, and rehabilitation in VR (Rinderknecht, Kim, Santos-Carreras, Bleuler, & Gassert, 2013).

However, despite the large variety of current applications and the future potential of these hand-tracking systems, there is little information regarding their actual performance, such as positional and angular accuracy and delay (Elliott et al., 2017). To address this, we introduce a methodological framework to compare marker-based (ground-truth) tracking to the performance of markerless tracking. Such methodology is similar to (Niehorster, Li, & Lappe, 2017), but here it is applied specifically to hand tracking. We use this framework to assess the performance of the Quest 2 hand-tracking system, which is one of the most popular and widespread examples of the use of this technology. In the rest of the paper, first, we introduce the methodological framework and its application, and then we present the outcome of the assessment. Finally, we provide an error-correction technique based on lens distortion to correct the Quest 2 positional errors (Park, Byun, & Lee, 2009).

## Methods

### Participants

Eight healthy volunteers (three female, age range, 25–48 years) were recruited from the University of Birmingham. All participants had a full range of arm and hand movements with normal or corrected-to-normal vision. This group of participants covered a range of hand sizes, with a measured circumference around the hand, ranging from 17.7*cm* to 22.2*cm*. The study was conducted according to the protocol approved by the STEM ethics committee of the University of Birmingham.

### Hardware

**Fig. 2:**
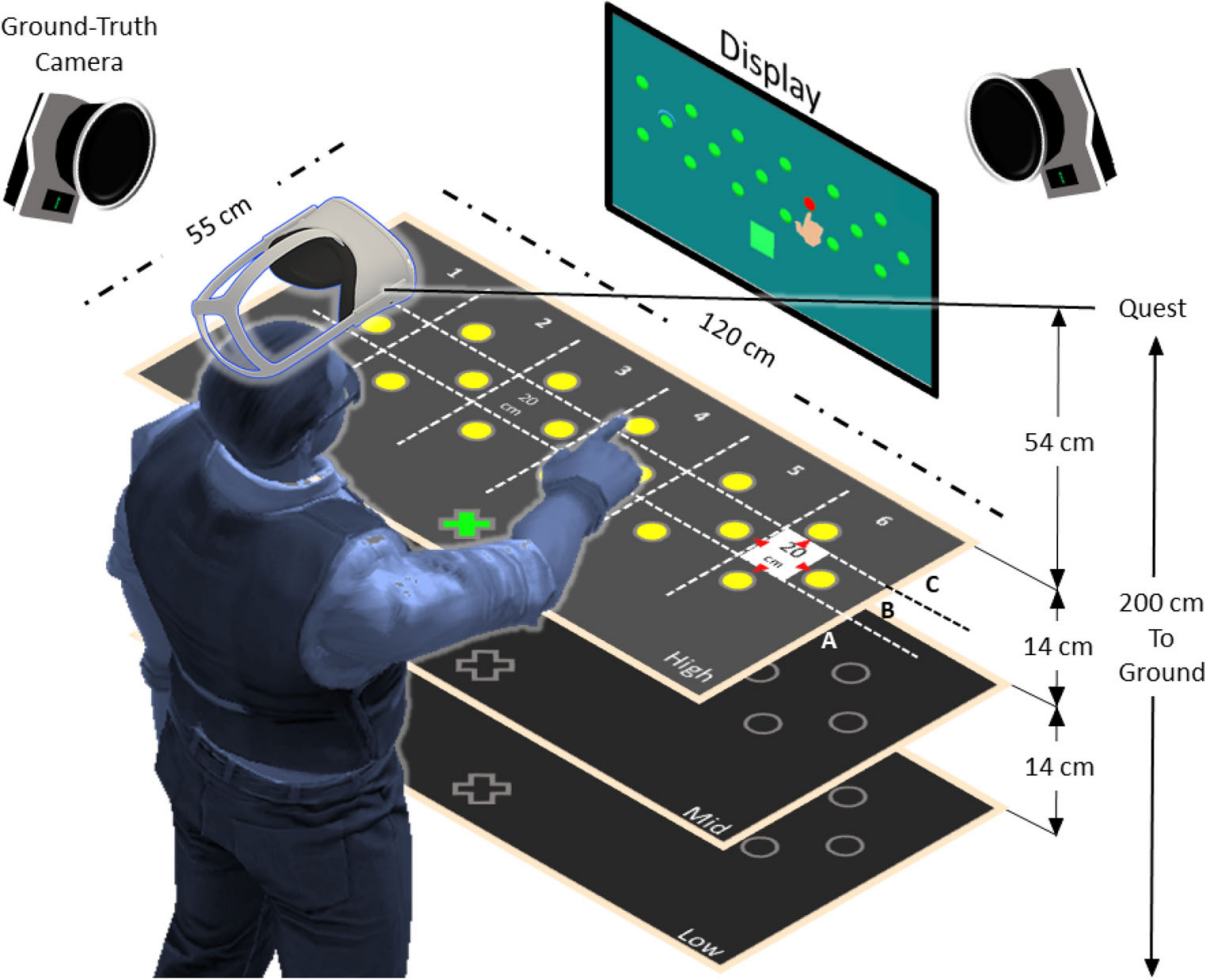
Experimental setup with the target panel, display, ground-truth cameras, and user standing under the Meta Quest 2 HMD. The targets (*yellow circles*) are separated into rows A, B, and C, and columns 1 to 6 with a total of 18 targets per each height. The *green cross* is the starting position

A Meta Quest 2 (the company was formerly named Facebook and now it is now named Meta) build version 33.0 head-mounted display (HMD) was rigidly held in a fixed position directly above the participant’s head but not blocking vision, with a downwards angle of 35^∘^ towards a table with 18 equally spaced infrared (IR) reflective marker targets of 2*cm* radius. The 18 targets were glued in a three rows (A, B, C) and six numbered columns (1–6) to a 120*cm* × 55*cm* × 1.5*cm* piece of acrylic sheet, Fig. 2. The grid of markers had a uniform spacing of 20*cm* in the three rows; the rows were 10*cm* apart and staggered to allow uniform sampling of the area within a rectangle of 110*cm* × 20*cm*. The target panel was supported by a height-adjustable table at the center of the motion capture space, and was used at three heights from the HMD, low (80*cm*), mid (66*cm*), and high (52*cm*), such that the volume sampled covered 0.528*m*^3^, Fig. 2. A VR-ready Windows laptop with a dedicated Nvidia GTX-1060 graphics card connected the motion capture cameras and the Quest. The capture space was illuminated evenly from all sides.

**Fig. 3:**
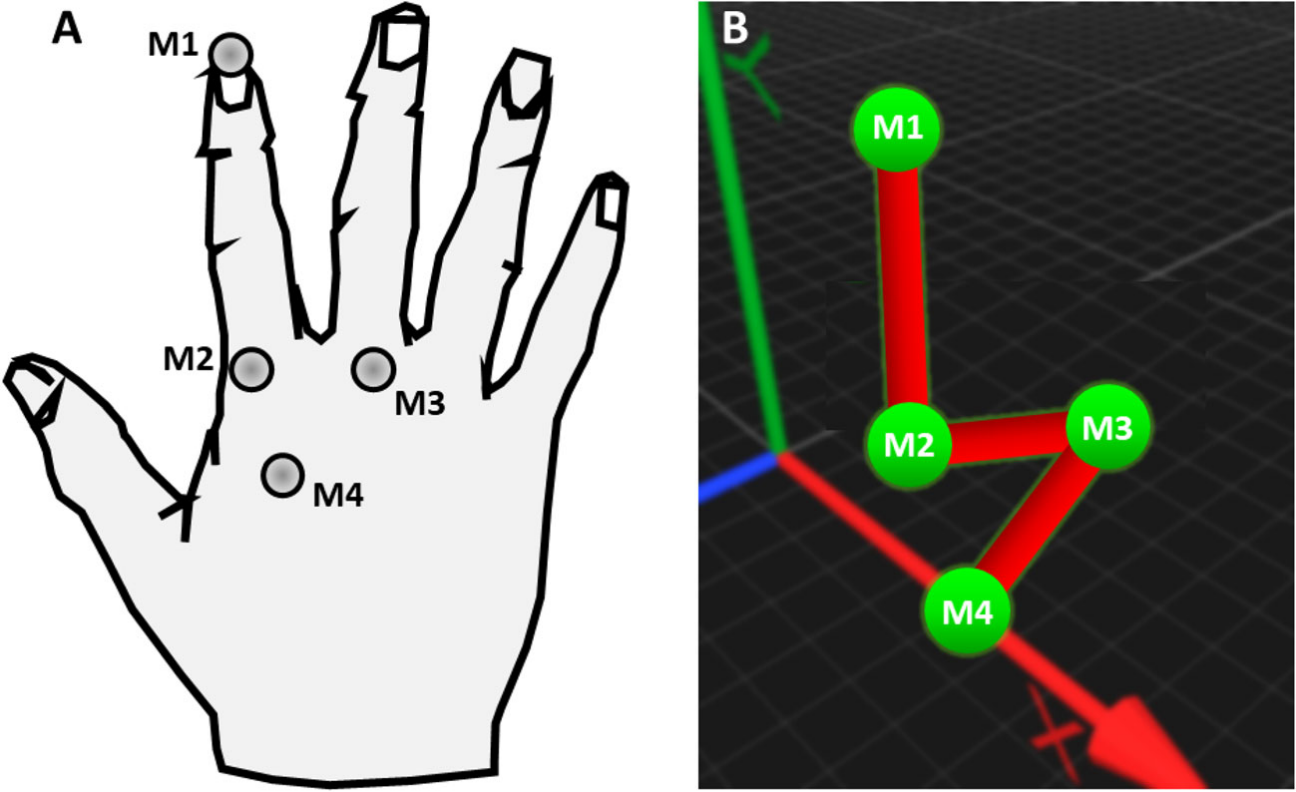
Hand model. A Infrared reflective markers (M1-M4) on the hand and fingers. B Corresponding computer model of the hand and fingers from Qualisys ground-truth tracking software (perspective view)

A set of 12 Oqus-350 cameras (Qualisys, 2021) were mounted on a frame on the ceiling and were aligned to cover the full tracking volume of the experiment. The cameras were used as our marker-based ground-truth measurement device. This setup achieves sub-millimeter positional accuracy (0.033*cm*) at a sampling rate of 240*Hz*, based on a calibration procedure performed before the experiment. Figure 3A shows how we arranged a set of four 0.2*cm* IR reflective markers on the participant’s hand and fingers to identify the individual markers on known anatomical finger locations via a computer hand model provided by the Qualisys software, Fig. 3B. This served as the ground-truth measure of fingertip position and timing.

### Software

A custom-built environment within the Unity game engine (Unity Technologies, *v*2021.1.9.*f*1) performed data recording and controlled the experiment’s progress, allowing the hand- and finger-tracking data to be recorded from both ground-truth and Quest hand-tracking systems synchronously at a sampling rate of 120*Hz*. To ensure that the sampling rate remained constant, a custom thread was implemented in C# with reference to the required variables of interest. The Unity-Oculus integration package and the Unity-Qualisys plugin allowed both systems to run from the same custom-built Unity environment. To use Quest native hand-tracking on the laptop, we used a Meta Link cable, which provided full access to all Quest functionality. We captured and stored the position of 18×3 target locations at the beginning of the experiment and used the Qualisys provided software (Qualisys Track Manager v2021.1) to align the ground-truth coordinate frame to that of the Quest-headset’s position, which we captured at the start of the experiment using a set of five ground-truth markers, Fig. 2. We confirmed this alignment by asking participants to use their index fingertip to reach the central target and the two most lateral targets at the beginning of each condition.

### Experiments

To measure positional accuracy and angular performance, two tasks were performed, a target-reaching and a hand opening-and-closing task. These tasks covered two of the most common motor control scenarios, which require hand position and orientation data as well as finger joint angles.

#### Target-reaching task

This task involved participants standing in front of the target panel and reaching one of the 18 targets of the current height (x3) in a random sequence. Each trial involved: 1. Placing the right index finger on the starting position, indicated by a sphere on the target panel closest to the participant, see the green cross in Fig. 2A; 2. Reaching to the highlighted target as indicated by a visual cue on the screen and by an auditory cue; 3. Maintaining fingertip position on the target for 1 s; 4. Returning to the starting position. Each target was repeated four times in each of the three blocked heights (low, medium, and high) and three tempos 80, 120, and 160 beats-per-minute (bpm), resulting in 648 trials per condition in total.

For each of the three tempos, a metronome dictated how fast the participant should move between the starting position and the target for the target-reaching task or how fast they should open and close their hand in the hand opening-and-closing task based on the temporal interval between the beats. In the target reaching task, the slowest to fastest tempos roughly corresponded to a range of velocities from 1.5 *ms*^− 1^ to 2.5 *ms*^− 1^. The upper velocity limit was chosen based on a pilot study, which found that for the Quest hand movements faster than about 3.2 *ms*^− 1^ resulted in unpredictable tracking behavior. Before each block of trials, participants had the opportunity to listen to several beats before executing the task and to practice the movements at the three different speeds, including receiving feedback from the experimenter.

#### Hand opening-and-closing task

Participants made a series of hand opening-and-closing movements for 2 min following one of the three tempos of 80, 120, and 160 bpm, while standing directly under the Quest, with the right hand raised chin-high and the palm rotated at a 45^∘^ angle towards their face for optimal hand and finger tracking of the Quest device. One trial of continuous opening-and-closing movements per tempo was recorded, lasting 60 s each.

### Data processing

**Fig. 4:**
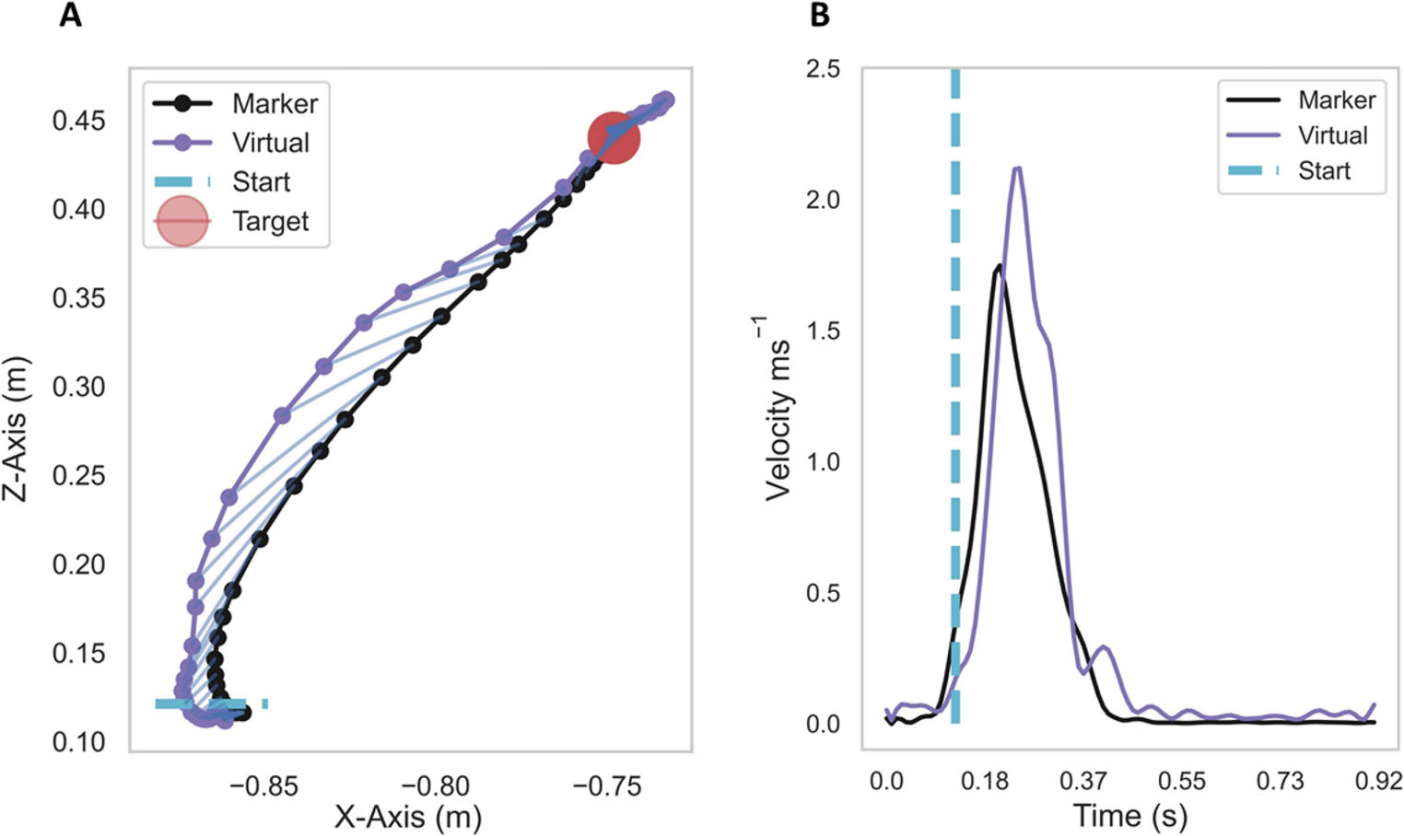
A: Example fingertip trajectory from the ground-truth marker and the Quest fingertip; B: The corresponding marker and virtual fingertip velocities. Data in these figures are for illustration only

Each trial recorded marker positions on the index finger and hand both from the ground-truth system and the Quest for this analysis. Figure 4A shows an example fingertip trajectory from the target-reaching task, together with its corresponding velocity, Fig. 4B. The dashed blue line in both figures indicates at which position and at what time point the fingertip leaves the starting region, following the start cue.

The following metrics were extracted for trials in the target-reaching task: maximum fingertip velocity, positional accuracy (i.e., error) and path offset between the real and virtual fingertips. The time delay (lag) between the virtual and real fingertips was computed for both the target-reaching and hand opening-and-closing tasks. Next, we describe how each metric was computed in more detail.

#### Delay

**Fig. 5:**
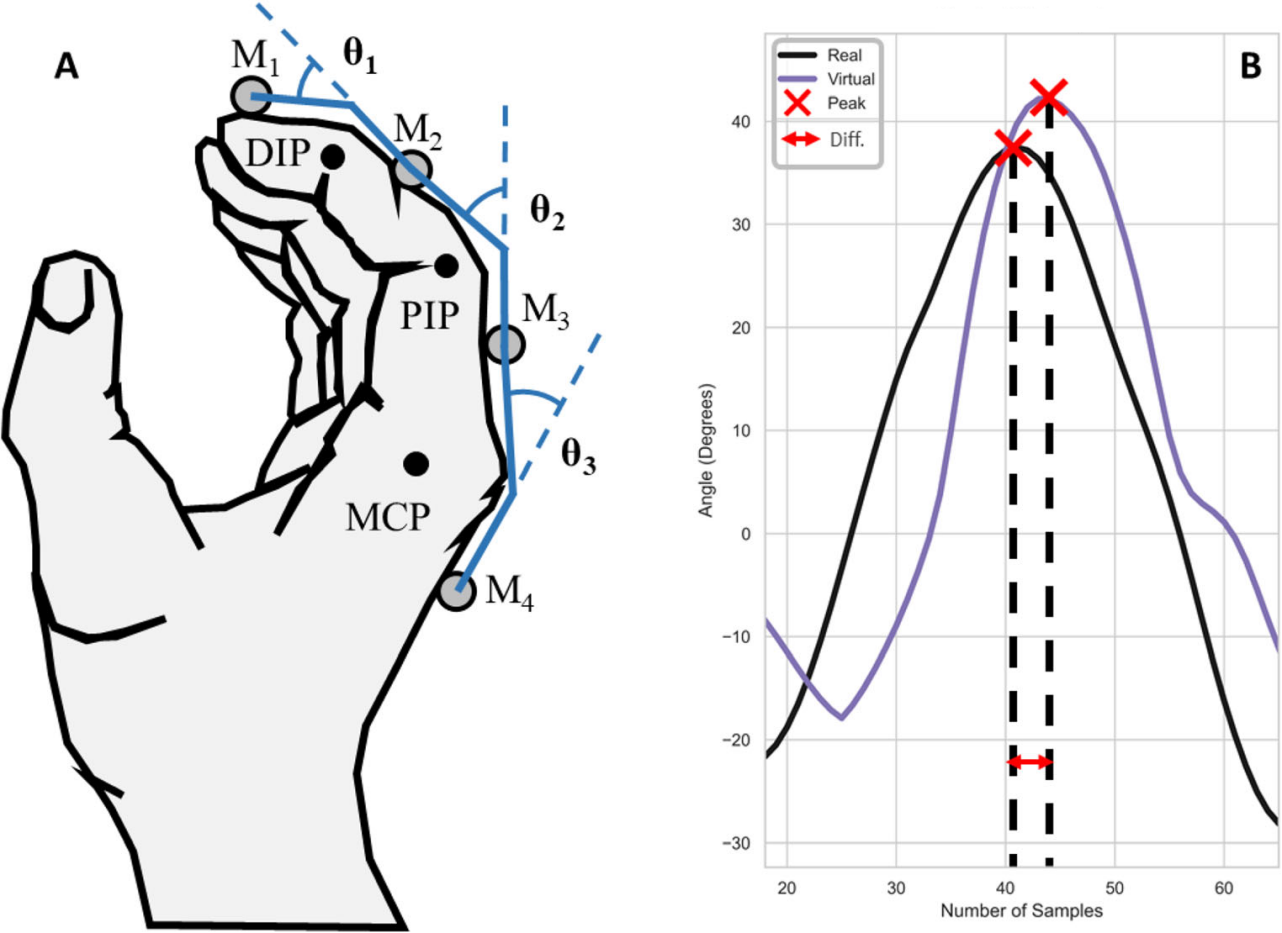
Hand opening-and-closing. A: Joint rotations showing the Distal (DIP), Proximal (PIP) and Metacarpo-phalangeal (MCP) joints of the index finger. B: Computing temporal delay between the virtual and real MCP joint angles using the peak angle (red crosses) as a metric for temporal alignment

The time delay was computed via the generalized cross-correlation tool using the positional and angular data from both Quest and ground-truth sensors (Droettboom & et al. 2003; Yamaoka, Scheibler, Ono, & Wakabayashi, 2019). We validated the delay results by randomly sampling from several trials using the separation between the two velocity peaks in time as the number of samples between the fingertip velocity and angular velocity peaks of the two sensors, through a peak detection tool (Jones, Oliphant, Peterson, & et al. 2001), see the two red crosses in Fig. 5B. To convert the number of samples back into a temporal delay (in milliseconds) the number of samples was multiplied by the sampling time, which is the inverse of the sampling rate:

1 $$ L = {{\varDelta}} f * {{\varDelta}} \nu_{0}, $$

where *Δ* f is the sampling time, and *Δν*_0_ is the difference in the number of samples between the ground truth and Meta Quest fingertip peak velocities.

Furthermore, we added the ground-truth delay of 7.0*ms* to our computed delay value to compute the final absolute delay. This ground-truth delay value was measured using a motion capture marker and a variable potentiometer to coupled marker movements with an analog voltage signal, see (Real-time latency tests of a Qualisys system in the Sensory-Motor Systems Lab at ETH, 2019)^1^.

#### Static positional accuracy

This metric was designed to estimate how the static positional accuracy of the Quest changed as a function of distance from the hand, without any consideration of movement speed or delay. To this end, we computed the Euclidean distance using the last 30 samples of Quest fingertip and ground-truth marker positions, following a one-second rest on the target to mitigate any temporal effects.

#### Path offset

To investigate the influence of movement speed i.e., the three tempo conditions, we computed a path offset metric, which was based on the Manhattan distance (D), Eq. 2. The distance between each sample was defined as the sum of the absolute differences between two vectors, in our case the trajectories of the ground truth and Quest fingertip:

2 $$ D(m,q) = \sum\limits_{i=1}^{n} |m_{i} - q_{i}|, $$

where (m,q) are the trajectory vectors for the ground-truth and Quest fingertip data, respectively.

To mitigate the effects of delay between the two tracking systems on this offset metric, the analysis shifted the Quest fingertip trajectory back in time by the average delay amount of 34.0 ms, as explained in Section “Delay”.

#### Angular error

For the hand opening-and-closing task, the Quest provided joint angles directly, which we compared to the joint angles computed from the ground-truth markers. As a result, we had to change the marker arrangement to a row along the index finger. Figure 5A shows the hand during the opening-and-closing cycle, where the three joint rotation angles between the four phalanges of the index finger are illustrated based on the four markers (M1-M4). The inverse of the cosine function computes the angle between each pair of adjacent markers, M1-M2, M2-M3, and M3-M4, resulting in three angular values for each of the three joints: distal (DIP), proximal (PIP) and metacarpo-phalangeal (MCP) of the index finger.

The time derivative of the joint rotations provides another metric, angular velocity error, which is computed from the average of the absolute difference between the real and virtual joint angle velocities for each of the joints, Fig. 5B. This metric is delay corrected, meaning the error computations takes into account the delay in angular velocity between the Quest fingertip and ground-truth marker by shifting the Quest data back in time by the computed delay.

#### Statistics

For each outcome metric, we used the R-Studio programming language (R-Studio v1.1456, R v3.61) together with the “lme4” statistics package to fit a generalized linear mixed-effects model (GLMM) using the three fixed effects (tempo, height and target location) and subject as a random factor. The default Gaussian regression function was used in all applications of the GLMM function using random intercepts. To report the resultant GLMM model the “Anova” function from the “car” statistics package was used, which provided the F-value, degrees of freedom and *p* values. For post hoc analysis, we applied pairwise least-squares means comparisons from the “multcomp” and “lsmeans” R statistics packages with sequential Bonferroni adjustments. We chose the GLMM model to account for non-normally distributed data, as was present in a pilot data set. Most metrics showed a beta distribution.

## Results

### Delay

During the reaching task, the Quest hand and finger tracking data showed an average delay of *M* = 38.0 *ms*, *SD* = 31.9 *ms* when compared to the ground-truth data. We have added the previously stated ground-truth delay of 7 *ms* to this figure to get the absolute delay of the Quest system of 45.0 *ms*. The GLMM showed that the delay was independent of height *F*(2,2478.9) = 1.1, *p* = 0.16, tempo *F*(2,22477.8) = 1.3, *p* = 0.08 and target location *F*(17,2477.1) = 11.5, *p* = 0.43.

### Static positional accuracy

**Fig. 6:**
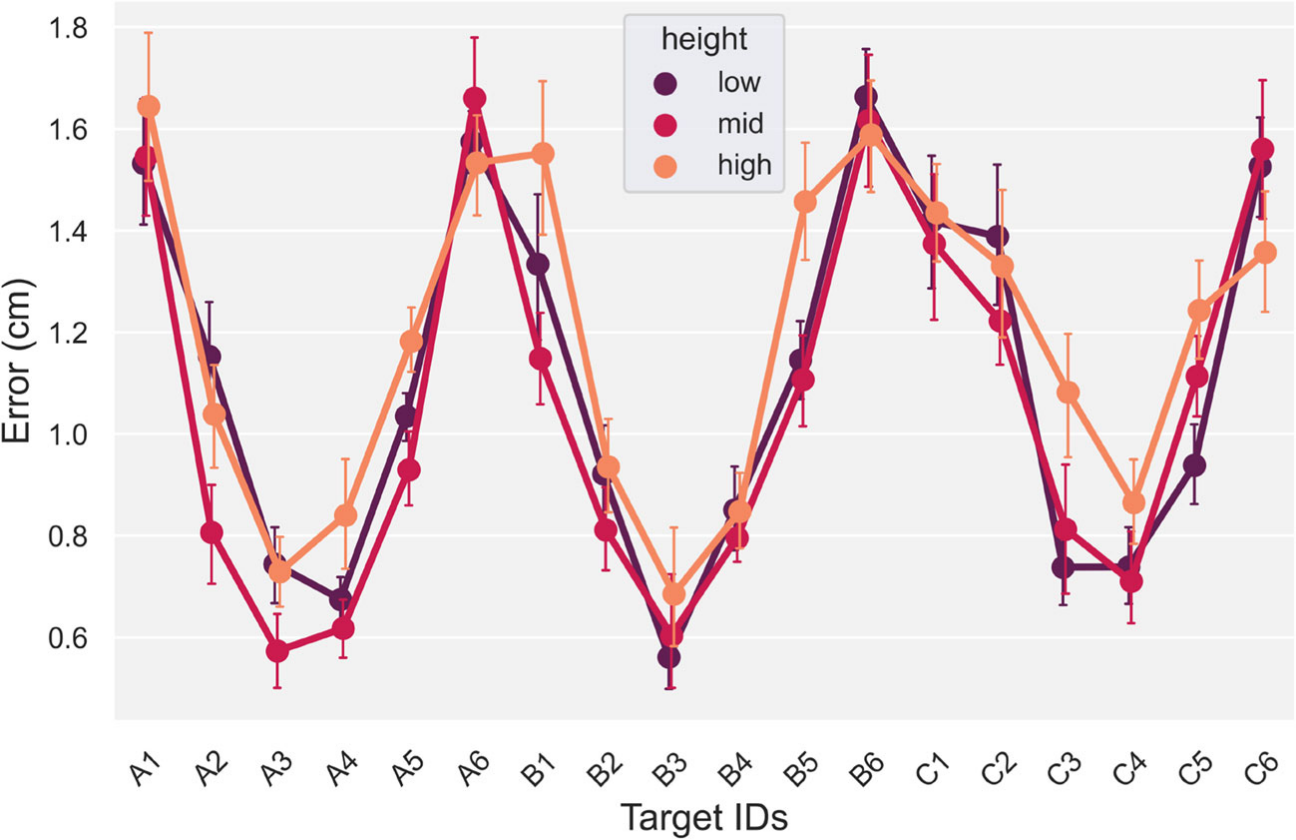
Positional error ± standard error of the mean, across the three heights and 18 targets, averaged across the three tempos

This error metric showed a characteristic pattern with larger static positional errors in the periphery of the Quest’s visual field, i.e., when the fingertip was farthest away and to the periphery of the HMD, Fig. 6. In contrast, height did not have a significant effect on positional accuracy, *F*(2,2477.5) = 1.3, *p* = 0.27. For this metric, we do not consider tempo as a factor, because we were only interested in static positional accuracy. Therefore, positional error was averaged across the three heights and tempos for the subsequent analysis. The GLMM showed a main-effect for planar target location on positional accuracy, *F*(17,2477.1) = 17.4, *p* < 0.001.

Specifically, a post hoc analysis showed peripheral targets, i.e., A1, A6, B1, B6 and C1, C6, had a significantly higher positional error (*M* = 1.3*cm*,*SD* = 0.46*cm*) compared to the positional errors measured for the central targets (targets 2 − 5 for rows *A* − *C*), (*M* = 0.79*cm*,*SD* = 0.39*cm*), ($t = 7.2, p < 0.01, {\eta _{p}^{2}} = 0.62$). There was a significant interaction between height and target, *F*(34,2477.1) = 2.3, *p* < 0.001. A subsequent post hoc analysis on this interaction showed the error for central-mid-level targets (*M* = 0.75*cm*,*SD* = 0.1*cm*) was significantly smaller, ($t = 8.2, p < 0.001, {\eta _{p}^{2}} = 0.54$), compared to peripheral targets at the low and high level (*M* = 1.3*cm*,*SD* = 0.1*cm*).

### Path offset: Trajectory error

**Fig. 7:**
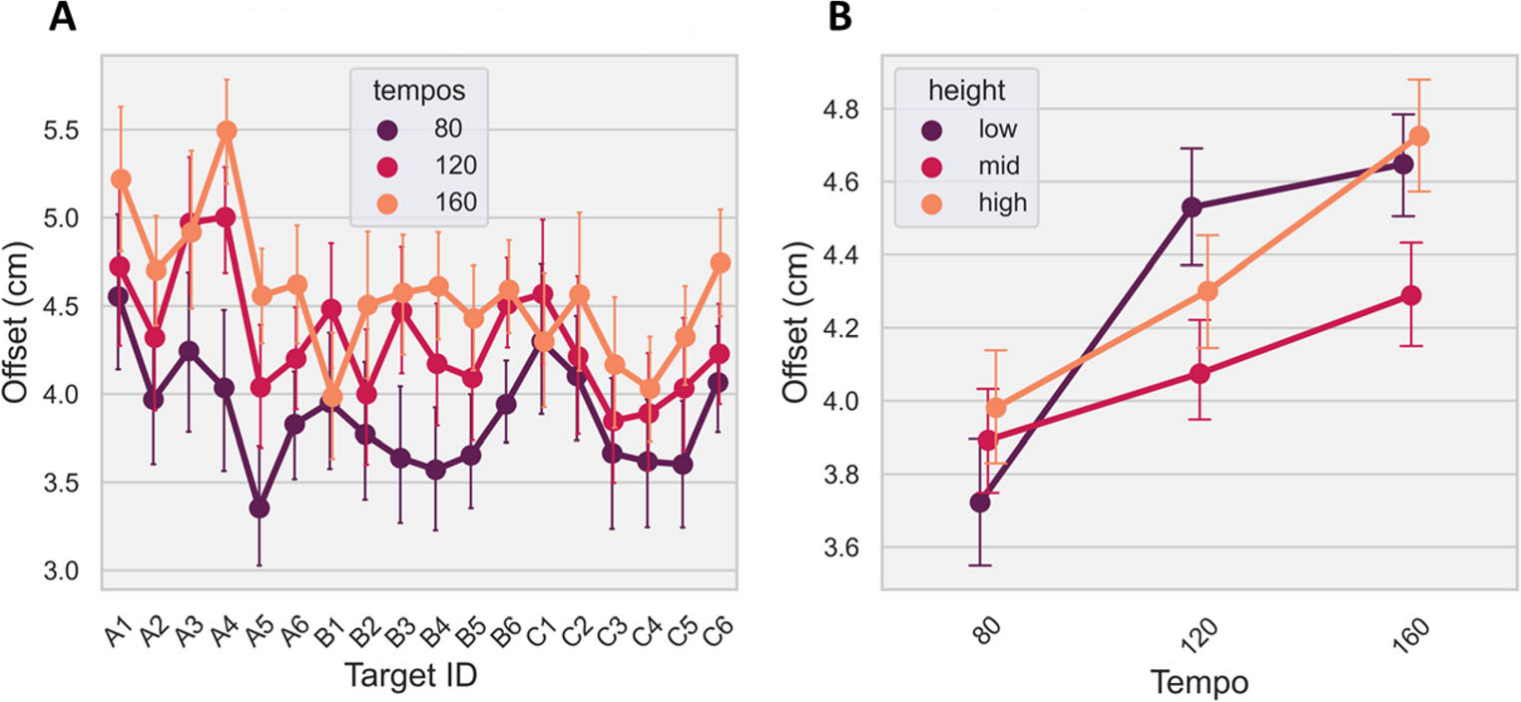
A: Path offset across the three tempos and 18 targets, averaged across the three heights; B: Path offset with standard error across the three tempos and heights, averaged across the targets. Error bars represent standard error of the mean

This error metric compared the Quest and ground-truth fingertip trajectories during movement from the starting position to the target on a sample-by-sample basis (i.e., error across a movement). As a result, the baseline delay between the two sensors was corrected by shifting the Quest data samples forward by 34.0 *ms* in line with the ground-truth data. This step ensured the measurements were taken between temporally synchronous samples and therefore they better represented the path offset. As shown in Fig. 7A, path offset was heavily influenced by the speed of the movement and the relative distance to the HMD. Specifically, a GLMM revealed a significant main effect for tempo (*F*(2,2478.0) = 60.9, *p* < 0.001), height (*F*(2,2479.2) = 11.8, *p* < 0.001) and target (*F*(17,2477.1) = 7.8, *p* < 0.001). In addition, there was a significant interaction between tempo and height (*F*(4,2477.6) = 6.2, *p* < 0.001) and marginal interactions between tempo and target (*F*(34,2477.0) = 1.4, *p* = 0.054) and height and target (*F*(34,2477.1) = 1.5, *p* = 0.057). Post hoc tests showed that the mid-layer height had a significantly lower path offset (less error), $t = 2.2, p < 0.05, {\eta _{p}^{2}} = 0.3$, compared to the other heights in the 120*bpm* (*M* = 4.1*cm*,*SD* = 1.2*cm*) and 160*bpm* (*M* = 4.3*cm*,*SD* = 1.2*cm*) tempo conditions but not for the 80-bpm condition, Fig. 7B.

### Angular error

**Fig. 8:**
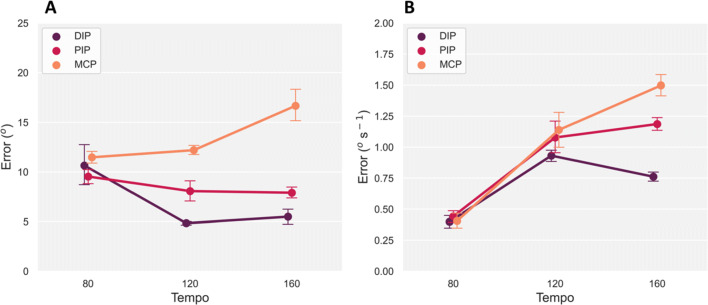
A: Angular error ± standard error of the mean, between ground-truth marker and Meta Quest averaged across the three joints (DIP, PIP and MCP). B: Corresponding angular velocity error with standard error between the groundtruth marker and Meta Quest sensors

The hand opening-and-closing task provided joint angular rotations for each pair of adjacent joints of the right-hand index finger via the ground-truth markers, Fig. 5A. We compared these angular estimates with the joint angle measures provided directly by the Quest hand tracking, including angular velocity error. Figure 8A shows angular error from the Quest changed with increasing rotation speed across all three joints, with error increasing for the MCP joint from 11.5^∘^ at the 80*bpm* level to 16.7^∘^ at the 160*bpm* tempo. In contrast, error decreased in the other two joints with increasing tempo. The GLMM analysis confirmed these observations with a main effect of tempo *F*(1,893.0) = 47.8, *p* < 0.001 and joint *F*(2,893.0) = 15.5, *p* < 0.001. There was also a significant interaction between tempo and joint, *F*(2,893.0) = 49.8, *p* < 0.001. A post hoc test showed interactions between these two factors. Angular error between the three joints at the 80*bpm* tempo level was not significantly different, ($t = 1.1, p = 0.95, {\eta _{p}^{2}} = 0.1$). However, for the 120*bpm* and 160*bpm* tempos, angular error was significantly different between the three joints, ($t = 10.4, p < 0.001, {\eta _{p}^{2}} = 0.73$), confirming the Quest’s joint angle estimates increased in error with faster rotational movements in the MCP joint. The overall average angular error across the three tempos and joints was *M* = 9.6^∘^, *SD* = 6.2^∘^.

Angular velocity error presented an increasing trend across the three tempos showing larger separation in error with faster rotational movements of each joint, Fig. 8B. A GLMM analysis confirmed this trend for tempo, with a main-effect, *F*(2,890.0) = 43.0, *p* < 0.001. The three joints were not significantly different in velocity error from one another, *F*(2,890.0) = 0.32, *p* = 0.73. However, there was a significant interaction between tempo and joint, *F*(4,890.0) = 20.5, *p* < 0.001. A post hoc analysis showed that the separation in velocity error between the PIP and DIP joints at the 160*bpm* tempo was significant, ($t = 5.3, p < 0.001, {\eta _{p}^{2}} = 0.31$). This significant interaction is also true for the other two joint pairs at the 160*bpm* tempo level, ($t = 12.5, p < 0.001, {\eta _{p}^{2}} = 0.74$).

### Error correction

**Fig. 9:**
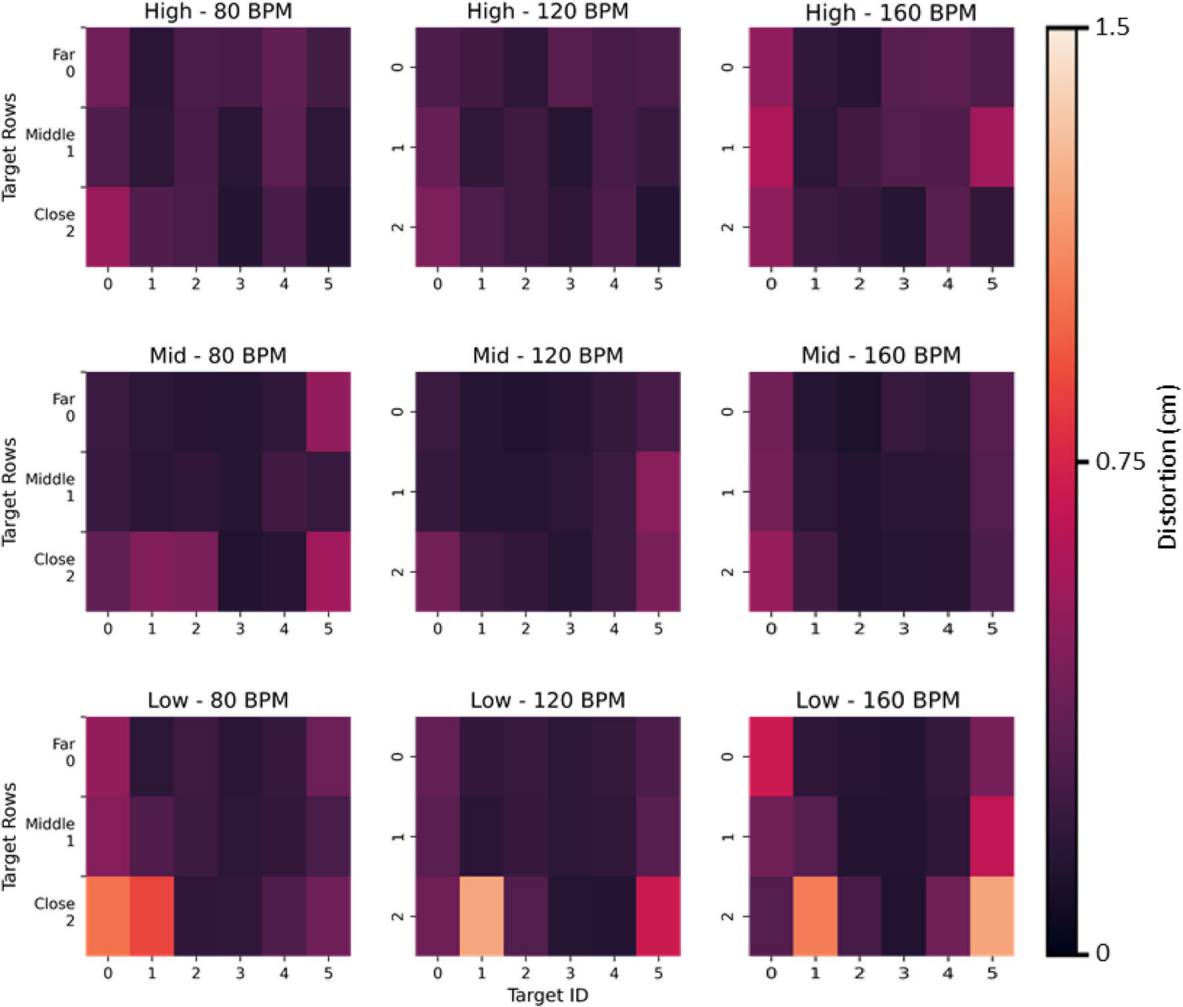
Heat-maps of the average positional error for a particular tempo and height across all participants. Each row of plots is for a particular target-panel height starting with the highest to lowest heights (top to bottom) and each column represents one of the three tempos (80, 120 and 160*bpm*) from left to right

The aim of this section is to present position-correction results from the previously-identified errors. Figure 9 illustrates the Quest’s static positional errors across its entire tracking volume, with accuracy worsening in the periphery of the visual field. This seems especially true for the lowest height i.e., the furthest distance between the target panel and the Quest HMD (bottom row in Fig. 9).

Together with the ground-truth data, these error matrices were used to construct a lens distortion model. To make an analogy, distortions in images taken by a camera are caused by imperfections in the camera’s lens, and to correct for these distortions we would need to characterize and reverse the offset at each location on the image. For a subset of the positional error data (80%), we defined a fifth-order polynomial lens distortion model (Eq. 3) and applied this to each of the three tempos and heights, with the four-trial average Quest and ground-truth positions (i.e., distorted and un-distorted positions, respectively) as input to the model:

3 $$ f(r) = r \cdot \left( 1 + \sum\limits_{n=1}^{N} p_{n} \cdot r^{n}\right) $$

where r is the raw positional data and *p*_*n*_ is the *n*^*th*^ parameter.

**Fig. 10:**
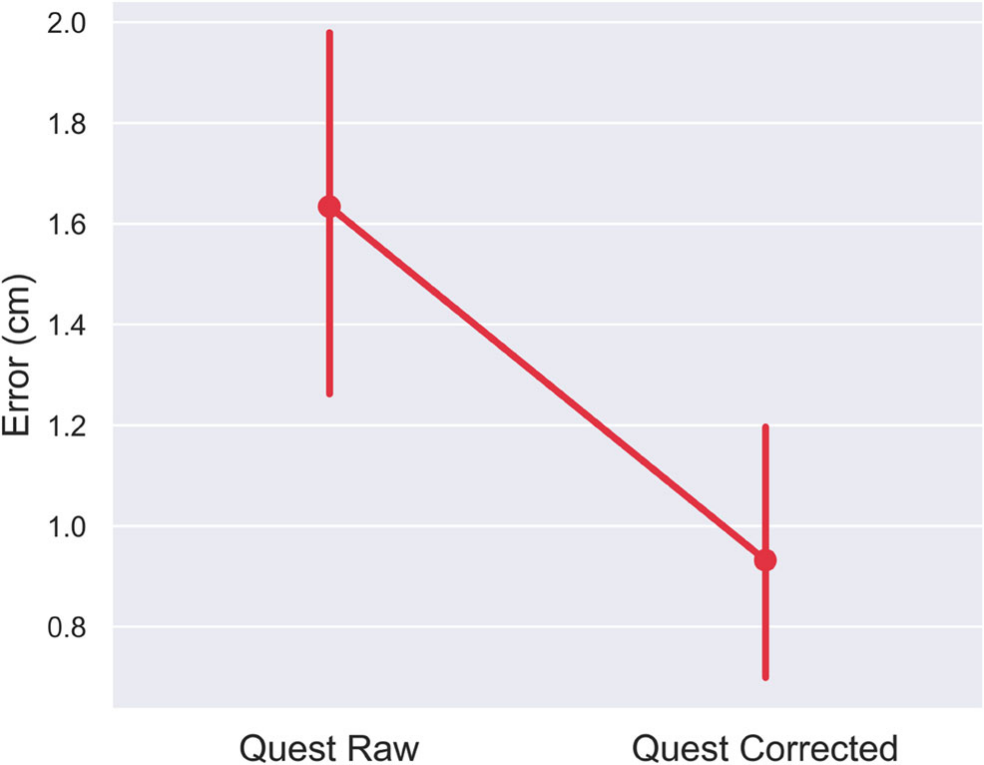
Correcting Meta Quest 2 tracking error ± standard error of the mean, using five lens correction parameters and a polynomial lens distortion model for target reaching across a large (120*cm* × 55*cm* × 80*cm*) tracking space. This set of data was not used when the parameters for the lens-correction model were computed

Based on this input, a least-squares optimization tool, (Jones et al., 2001), computed a set of five lens correction parameters by minimizing the error between the Quest’s raw input and the ground-truth positions through an iterative optimization procedure, Table 1. A paired *t* test showed these five parameters were sufficient to improve the positional error of the remaining 20% of unseen raw position data, as this resulted in corrected position data that was significantly closer to the ground-truth (*t*(3.1),*p* < 0.001) as opposed to the raw Quest positions, Fig. 10.

**Table 1:** Lens distortion correction parameters for reconstructing the Meta Quest 2 positional tracking errors

Param 1	Param 2	Param 3	Param 4	Param 5
19.58	− 237.14	1058.94	− 2072.77	1502.66

## Discussion

This article presents a novel methodological framework to assess the accuracy of VR optical hand- and finger-tracking systems, such as the popular Meta Quest 2 VR headset. Furthermore, we present a novel approach to account and correct for tracking errors based on a lens-distortion model. The results show a set of useful metrics such as accuracy (static measure of positional error), path offset (trajectory error during motion), angular error and temporal delay. We believe these metrics could be highly informative when deciding whether to use such hand-tracking systems within environments that require high spatial and temporal accuracy (i.e., motor control/learning research).

The method presented here provides clear quantitative evidence to show that static positional errors are largely affected by the distance between the hands and fingers from the central view area of the headset, with larger positional errors further away from the center. We speculate this is most likely due to a combination of factors related to the headset’s camera lens distortion-correction model and the camera’s resolution. Fingers in the periphery of vision are distorted to a larger degree compared to the center, resulting in the Quest’s hand tracking model underestimating the finger’s true position. Future work could focus the analysis on the directionality of the positional errors in order to confirm that peripheral position estimates are indeed overestimated by the model compared to the central position estimates.

For path offset, height, tempo, and target location all had a significant impact on this cumulative metric, because in any given trial hand motion could cover a large tracking space (i.e., nearer or further away from the headset), and with one of three motion speeds. Faster movements resulted in larger offsets. In this case, it is likely the Quest’s pre-trained machine-learning model receives fewer frames of the hand in rapid motion, such as in the 160*bpm* condition. Compared to the 80*bpm* condition, these fewer frames of the hand could result in fewer data points for the algorithm to accurately estimate key-points around the hand and fingers (Han et al., 2020).

However, because the Quest uses a proprietary hand-tracking algorithm, it is difficult to know for certain which computational stage would be most affected by rapid hand movements. Google’s open-source, camera-based MediaPipe hand-tracking algorithm provides insight into the inner-workings of the Quest hand-tracking algorithm, who show that their machine-learning algorithm is based on a set of assumptions about skin tones, textures, hand sizes, camera model, and hand movement parameters, (Zhang et al., 2020). Based on this, it is fair to assume that the Quest’s pre-trained algorithm is also based on a similar set of assumptions. An equally viable argument for why tracking performance decreases during fast motions could be made based on the other stages of the algorithm, such as the hand detection or application of inverse kinematics stages. In our case, another impact-full test parameter could be related to skin tones. Although our design covered a relative diverse set of hand sizes, we only recruited participants with skin tones in the light range. This is an important area to consider when planning to use the Quest hand tracking for research-related purposes.

The overall average angular error was 9.6^∘^, which was differentially affected for each joint with increasing tempo. We did find that increasing rotational movements of the MCP joint was significantly increased angular errors. This could be due to self-occlusions, where the other finger phalanges block the direct visual line of sight of the MCP joint when closing the hand in a palm-facing direction. The MCP joint is occluded and the algorithm has to rely on previous joint estimates, which could be incorrect, or estimate the occluded joint angle based on other parameters, such as the other visible joints and the overall hand pose, all of which would be a less accurate estimate of the actual joint angle. However, this speculation requires further testing with a new experimental design, such as performing hand opening-closing at different viewing angles relative to the Quest’s cameras.

Angular velocity error was significantly lower in the low-speed condition (80*bpm*) compared to the 120*bpm* tempo but not the 160*bpm* tempo condition. One explanation for this is self-occlusions of the proximal joint by the back of the hand when making a fist during the hand opening-and-closing task. This interrupts the stream of images of the hand to the algorithm, which pushes the estimates to rely on a sparser data set to estimate joint angles, resulting in larger errors. To test this hypothesis, more hand opening-and-closing task data are required at different viewing angles relative to the headset so that self-occlusions can be avoided.

Delay is one of the most important metrics in gaming, research and robotics because it determines the limits of applicability. The results showed an average absolute delay of 45.0 *ms* in the Quest hand-tracking data, which was unaffected by motion speed or location of the hand relative to the headset. Therefore, a major component of this delay is most likely caused by the headset’s optical sensors, processor and wireless communications capabilities. In contrast, others have found a larger latency of the order of 81*ms* for the Quest v1 video-pass-through, as used for augmented reality applications, (Gruen et al., 2020). Although this study did not look at hand- and finger-tracking latencies, the question about why this latency figure is twice as high as what we have found is still valid. To account for these large delay values, we highlight two important differences between (Gruen et al., 2020) and our reported delay: 1) video passthrough does not have latency compensation and 2) we used the more advanced Quest v2 headset with a faster processor and upgraded software.

Furthermore, as delay is a hard limit, the temporal delay must be taken into account when using the Quest within any environment in which the tracking data is being used to assess motor performance.

To expand the usable range of the Quest hand-tracking for static positional estimates, we applied a lens-distortion model across the entire tracking space to correct errors. With only five parameters, it was possible to correct positional errors across the Quest’s tracking volume. While this appears to be a powerful technique to correct the spatial errors observed in the hand-tracking data, further analysis is required to test the limits of this approach. For example, at what level of tracking error does the reconstruction break down by providing worse results than the raw tracking data from the optical machine-vision system?

Despite the positional errors and the presence of lag, there is little reporting of clear discrepancies between actual and virtual hand movements within VR apps that use markerless hand tracking. This indicates that within an immersive environment, these discrepancies may not be noticeable. However, when deciding whether to use these hand-tracking systems for experimental paradigms, researchers should be aware of these errors and evaluate the consequences for their research goals. For example, in the field of motor control, such temporal and spatial measurement errors may be highly undesirable during dynamic tasks (where the errors are magnified) such as motor adaptation, (Krakauer, 2009), online tracking, (Foulkes & Chris Miall, 2000) and grasping, (Castiello, 2005) but less so during static tasks (where the errors are minimized) such as proprioceptive realignment, (Block & Bastian, 2011). In addition, due to the reduced accuracy requirements, research focusing on social interaction may be less affected than tasks involving object manipulation. Similarly, if the emphasis is on rehabilitation and encouraging the use of the hands, then the consequences of accurate measurement may not be of such importance, in comparison to the immersion in the task. The results presented here enable researchers to make informed choices and also follow our methodology to correct some of these errors. Our results also enable certain recommendations when planning experiments or tasks, such as keeping the hands nearer the center of the tracked volume directly in front of the HMD and emphasizing postures which do not occlude the fingers. The framework detailed here also stands as a repeatable methodology to measure the hand-tracking accuracy of other devices and future generations of XR HMDs.

In summary, this article provides a robust methodological approach for assessing the temporal and spatial accuracy of VR-based markerless hand-tracking systems. Analysis of the Meta Quest 2 indicated clear temporal and spatial limits of the device for tracking hand-based movements, which either need to be adjusted for or taken into account when making conclusions based on the data.

## Open Practice Statement

All data and materials for all experiments are available at this-repository^2^ and none of the experiments were preregistered.

## Footnotes

1 shorturl.at/aqrwx

2 https://github.com/DiarKarim/MetaOculusQuestPerformance.git
